# Mediterranean dietary pattern and depression: the PREDIMED randomized trial

**DOI:** 10.1186/1741-7015-11-208

**Published:** 2013-09-20

**Authors:** Almudena Sánchez-Villegas, Miguel Angel Martínez-González, Ramón Estruch, Jordi Salas-Salvadó, Dolores Corella, Maria Isabel Covas, Fernando Arós, Dora Romaguera, Enrique Gómez-Gracia, José Lapetra, Xavier Pintó, Jose Alfredo Martínez, Rosa María Lamuela-Raventós, Emilio Ros, Alfredo Gea, Julia Wärnberg, Lluis Serra-Majem

**Affiliations:** 1 Biomedical Research Center Network on Obesity and Nutrition (CIBERobn) Physiopathology of Obesity and Nutrition, Institute of Health Carlos III, Madrid, Spain; 2 Department of Clinical Sciences, University of Las Palmas de Gran Canaria, PO Box 550, 35080 Las Palmas de Gran Canaria, Spain; 3 Department of Preventive Medicine and Public Health, University of Navarra, Pamplona, Spain; 4 Department of Internal Medicine, Institut d’Investigacions Biomediques August Pi Sunyer (IDIBAPS), Hospital Clinic, University of Barcelona, Barcelona, Spain; 5 Human Nutrition Unit, IISPV, Universitat Rovira i Virgili, Reus, Spain; 6 Department of Preventive Medicine, University of Valencia, Valencia, Spain; 7 Lipids and Cardiovascular Epidemiology Research Unit, Institut Municipal d’Investigacio Medica (IMIM), Barcelona, Spain; 8 Department of Cardiology, University Hospital Txagorritxu, Vitoria, Spain; 9 Research Unit, University Hospital Son Espases, Palma de Mallorca, Spain; 10 School of Public Health, Imperial College London, London, UK; 11 Department of Preventive Medicine, University of Malaga, Malaga, Spain; 12 Department of Family Medicine, Primary Care Division of Sevilla, Centro de Salud San Pablo, Sevilla, Spain; 13 Lipids and Vascular Risk Unit, Internal Medicine, Hospital Universitario de Bellvitge, Hospitalet de Llobregat, Barcelona, Spain; 14 Department of Nutrition and Food Sciences, Physiology and Toxicology, University of Navarra, Pamplona, Spain; 15 Nutrition and Food Science Department–XaRTA, INSA, University of Barcelona, Barcelona, Spain; 16 Lipid Clinic, Department of Endocrinology and Nutrition, Hospital Clınic, Barcelona, Spain; 17 Institut d’Investigacions Biomediques August Pi Sunyer (IDIBAPS), Hospital Clınic, Barcelona, Spain

**Keywords:** Mediterranean diet, Depression, Trial, Primary prevention, Nuts, Olive oil, Low-fat, Diabetes

## Abstract

**Background:**

A few observational studies have found an inverse association between adherence to a Mediterranean diet and the risk of depression. Randomized trials with an intervention based on this dietary pattern could provide the most definitive answer to the findings reported by observational studies. The aim of this study was to compare in a randomized trial the effects of two Mediterranean diets versus a low-fat diet on depression risk after at least 3 years of intervention.

**Methods:**

This was a multicenter, randomized, primary prevention field trial of cardiovascular disease (Prevención con Dieta Mediterránea (PREDIMED Study)) based on community-dwelling men aged 55 to 80 years and women aged 60 to 80 years at high risk of cardiovascular disease (51% of them had type 2 diabetes; DM2) attending primary care centers affiliated with 11 Spanish teaching hospitals. Primary analyses were performed on an intention-to-treat basis. Cox regression models were used to assess the relationship between the nutritional intervention groups and the incidence of depression.

**Results:**

We identified 224 new cases of depression during follow-up. There was an inverse association with depression for participants assigned to a Mediterranean diet supplemented with nuts (multivariate hazard ratio (HR) 0.78; 95% confidence interval (CI) 0.55 to 1.10) compared with participants assigned to the control group, although this was not significant. However, when the analysis was restricted to participants with DM2, the magnitude of the effect of the intervention with the Mediterranean diet supplemented with nuts did reach statistical significance (multivariate HR = 0.59; 95% CI 0.36 to 0.98).

**Conclusions:**

The result suggest that a Mediterranean diet supplemented with nuts could exert a beneficial effect on the risk of depression in patients with DM2.

**Trial registration:**

This trial has been registered in the Current Controlled Trials with the number ISRCTN 35739639

## Background

Unipolar depression affects more than 151 million people worldwide, is a leading cause of years of healthy life lost as a result of disability (years lost to disability; YLD) [1], and is projected to be the leading cause of disability-adjusted life years lost (DALYs) in 2030 [2]. However, little research has been performed on the modifiable risk factors of unipolar depression that can provide a means for its effective prevention. Whereas the role of diet in other chronic conditions with a high burden of disease such as cardiovascular disease (CVD) has been extensively studied, the role of diet in the prevention of mental disorders such as depression is an interesting field that has only emerged in the past few years [3].

Most of the epidemiological studies carried out to date have analyzed the relationship of individual food groups and nutrients (such as folic acid or omega-3 fatty acids) with the risk of depression [4-6]. Only a few epidemiological studies, most of which had a cross-sectional design, have analyzed the role of overall dietary patterns on depression risk [7-13]. A healthy dietary pattern such as the Mediterranean diet (hereafter referred to as MD) was uniquely associated with a lower risk of depression or depressive symptoms in two observational prospective studies [14,15].

However, one of the most important limitations in observational epidemiology is to obtain adequate control of confounding. This is a key methodological issue because potential effects of dietary patterns on depression could be explained in part by the co-occurrence of other lifestyle-related and sociodemographic factors, or by medical conditions closely related to the adherence to a particular dietary pattern.

This issue could be solved by carrying out large randomized primary prevention trials with interventions based on changes in the overall food pattern. The PREDIMED (Prevención con Dieta Mediterránea) trial was a nutritional intervention study designed to assess the role of the MD in the primary prevention of CVD [16], and provided an ideal setting to investigate the effect of the MD on the risk of developing depression. Thus the aim of this analysis was to assess the effects of two Mediterranean diets (MD supplemented with extra virgin olive oil (MD-EVOO), and MD supplemented with mixed nuts (MD-nuts)) on depression risk, in comparison with a low-fat control diet.

## Methods

### Study population

This study was conducted within the frame of the PREDIMED trial. The design of the PREDIMED trial has been reported in detail elsewhere [17]. Briefly, the PREDIMED trial was a large, parallel-group, multicenter, randomized controlled clinical trial that aimed to assess the effects of a Mediterranean-type diet on CVD. The study population was composed of men aged between 55 and 80 years and women aged between 60 and 80 years with no previously documented CVD, but who were at high cardiovascular risk. Inclusion criteria were either diabetes mellitus type 2 (DM2) or at least three of the following cardiovascular risk factors: current smoking, hypertension (HTA), low-density lipoprotein (LDL) cholesterol >4.110 mmol/l, high-density lipoprotein (HDL) cholesterol <1.034 mmol/l, overweight/obesity, or a family history of premature coronary heart disease.

**Figure 1:**
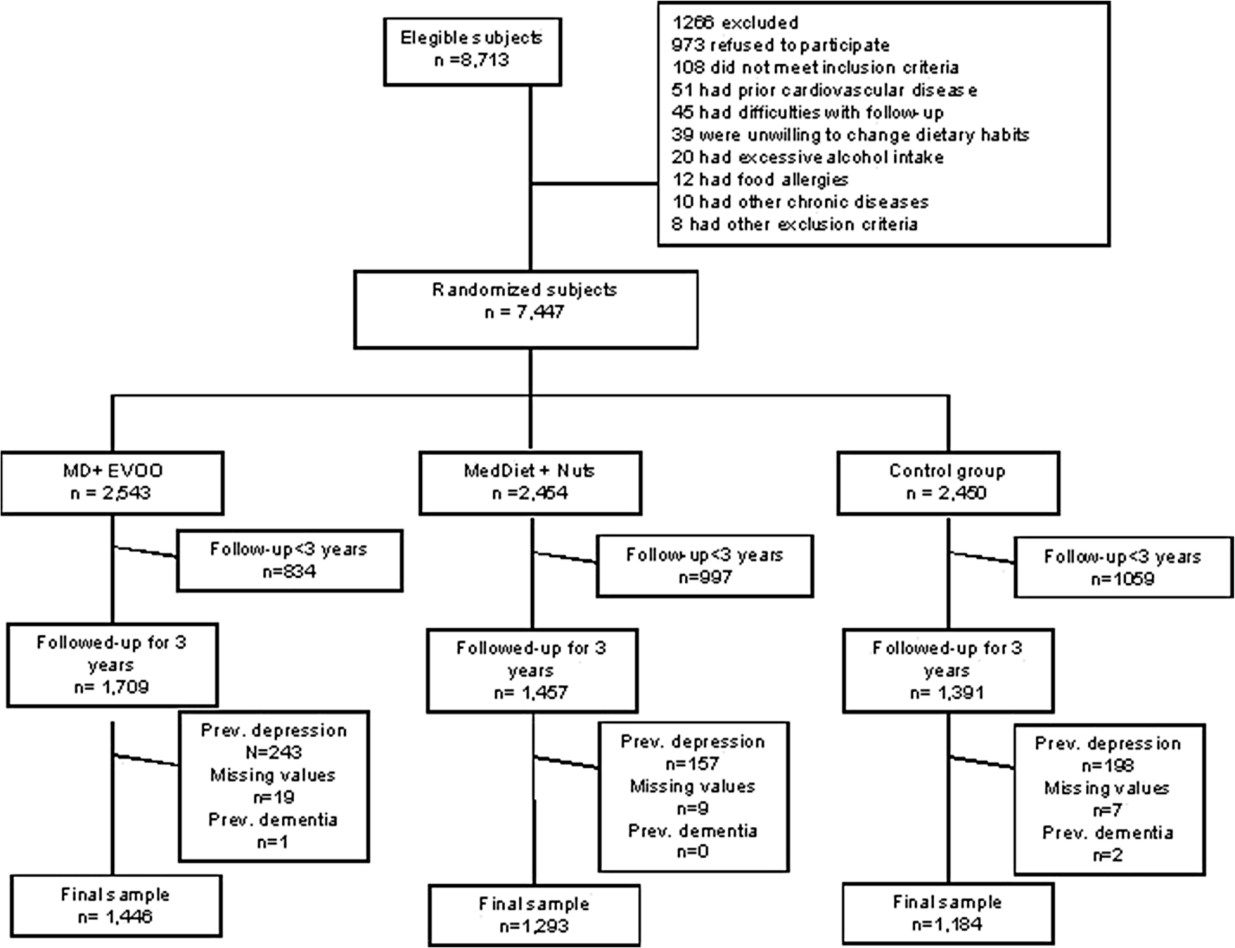
Profile of the PREDIMED (Prevención con Dieta Mediterránea) field trial.

Recruitment of participants took place in primary care centers affiliated with 11 Spanish teaching hospitals between October 2003 and June 2009. In total, 7,447 participants were recruited. Of these 1,618 subjects who reported prevalent depression, previous history of depression, or use of antidepressant drugs at baseline were excluded. Participants with a follow-up of less than 3 years (n=1,870) were also excluded in order to minimize the influence of possible reverse causation from the presence of undiagnosed disease at baseline, as were those with missing data for several covariates (n=33), and those with dementia (n=3). Thus, the final sub-sample consisted of 3,923 participants (Figure 1).

### Ethics

All participants provided written informed consent, and the protocol was approved by the institutional review boards of the participating centers, in accordance with the Declaration of Helsinki.The institutional review board of the Hospital Clínic (Barcelona, Spain), which is accredited by the Department of Health and Human Services and regulated by the Federal wide Assurance for the Protection of Human Subjects of International (Non-US) Institutions (number 00000738), approved the study protocol on July 16, 2002. This trial was registered with the Current Controlled Trials (number ISRCTN 35739639).

### Study interventions

Participants were randomly assigned, in a 1:1:1 ratio, to one of the three PREDIMED dietary interventions: the low-fat diet (control group), the MD-+EVOO, and the MD-+nuts by a nurse at each center. The study nurses were independent of the nurse staff of the primary care center, and therefore, were not involved in the usual clinical care of participants, their primary and exclusive role was to collect the data for the PREDIMED trial. Randomization was performed centrally by means of a computer-generated random-number sequence. The primary care physicians did not participate in the process of randomization.

The two groups allocated MDs received intensive education to follow the MD and supplemental foods at no cost. EVOO (1 l/week) was provided to the MD-EVOO group, and 30 g/day of mixed nuts (15 g walnuts, 7.5 g hazelnuts and 7.5 g almonds) to the MD-nuts group. The control group did not receive education on the MD, but was instead given advice to follow a low-fat diet, including recommendations to reduce all types of fat intake, from both animal and vegetable sources, in accordance with American Heart Association guidelines [18]. To encourage adherence, small non-food gifts, such as kitchenware, tableware, aprons, or shopping bags, were given. Energy restriction was not advised and physical activity was not promoted for any of the groups.

A behavioral intervention promoting the MD was implemented in both MD groups, as described previously [16,19]. In brief, an initial assessment of individual scores of adherence was carried out using a validated 14-item questionnaire [20], and based on this, 25 dietitians gave personalized dietary advice to participants randomly assigned to either of the MD groups, with the aim of improving participants’ scores on the questionnaire. Recommendations included, among others: 1) abundant use of olive oil for cooking and dressing; 2) increased consumption of fruit, vegetables, legumes, and fish; 3) reduction in total meat consumption, recommending white meat instead of red or processed meat; 4) preparation of homemade sauce with tomato, garlic, onion, and spices with olive oil to dress vegetables, pasta, rice, and other dishes; 5) avoidance of butter, cream, fast food, sweets, pastries, and sugar-sweetened beverages; and 6) (for alcohol drinkers) moderate consumption of red wine.

At baseline and every 3 months, dietitians conducted both individual interviews and group sessions with a maximum of 20 participants, separately for each group. Sessions consisted of informative talks and delivery of written material with detailed descriptions of typical foods for each dietary pattern, seasonal shopping lists, meal plans, and recipes.

### Outcome

An incident case of depression was defined as a diagnosis of depression made by a physician and reported by participants in any of the follow-up interviews, or a positive report of habitual use of antidepressant drugs. Only outcomes occurrring between October 1, 2006, and December 1, 2010, were included in the analyses.

### Covariates

At baseline and at follow-up once yearly, a validated semi-quantitative 137-item food frequency questionnaire [21,22] was administered by trained dietitians to determine energy and nutrient intake, using Spanish food composition tables [23]. Alcohol intake was also ascertained through use of this questionnaire.

Physical activity was measured at the baseline visit and at follow-up every year using the validated Spanish version of the Minnesota Leisure Time Physical Activity Questionnaire [24]. Other lifestyle-related variables such as smoking, health conditions (for example, prevalence of any one of a number of chronic diseases), and sociodemographic variables (for example, sex, age, educational level, marital status) were assessed by a general questionnaire based on 47 items, as described in detail previously [16,17]. In addition, anthropometric variables such as height and weight were also measured. Body mass index (BMI) was defined as weight (in kilograms) divided by the height (in meters) squared.

### Statistical analysis

Primary analyses were performed on an intention-to-treat (ITT) basis. Cox regression models were used to assess the relationship between the nutritional intervention groups and the incidence of depression. Hazard ratios (HR) and 95% confidence interval (95% CI) were calculated using the control group as the reference category. Participants contributed to the follow-up period up to the date of death, diagnosis of depression, or December 1, 2010, whichever came first. Potential confounders included as covariates in the multiple-adjusted Cox regression models were: age, sex, recruiting center (11 centers), BMI (normal, overweight, obese), smoking (never-smoker, current smoker, and ex-smoker), physical activity during leisure time (quartiles), educational level (primary, secondary, or university), marital status (married, others), alcohol intake (g/day), and total energy intake (kcal/day) and prevalence of various chronic diseases (cancer, DM2, HTA, hypercholesterolemia, fractures, Parkinson disease (PD), and chronic bronchitis) at baseline.

Several sensitivity analyses were conducted by alternative Cox regression analyses after 1) excluding late cases of depression (after 6 years of follow-up); 2) including only participants with DM2, HTA, or hypercholesterolemia; 3) excluding those participants with a history of limiting diseases (fractures, PD, and chronic bronchitis) or cancer; and 4) excluding those centers with lower retention rate.

In addition, in the sensitivity analyses those participants whom the PREDIMED team had not been able to contact for at least 2 years and who did not have a diagnosis of incident depression were considered as participants with a missing value for the outcome. In such cases, the analyses were also repeated after multiple imputation for these missing data [25].

Finally, in addition to the previous ITT analysis, the analyses were also repeated using a per-protocol analysis [26]. After 3 years of follow-up, participants completed the 14-item MD adherence questionnaire [20]. Three categories of adherence after 3-year follow-up were created: high (≥10 points in the score); medium (8 to 9 points) and low (<8 points) adherence to the MD. Those participants with a 3-year Mediterranean score of less than 8 points were considered as the reference category in the Cox regression models. The results were adjusted for age, sex, intervention group, recruiting center, smoking, educational level, marital status, and prevalence of various chronic diseases at baseline, plus BMI, alcohol intake (except wine - this item was included in the 14-item MD adherence questionnaire), energy intake, and physical activity after 3 years of follow-up.

All P-values were two-tailed, and *P*<0.05 was considered significant. Analyses were performed using the software programs SPSS (version 19; SPSS Inc., Chicago, IL, USA) and STATA (version 12.1; StataCorpLP, College Station, TX, USA).

## Results

The main characteristics of the participants in each nutritional intervention group are presented in Table 1. The groups were well balanced with respect to most relevant variables. Participants in the control group were less physically active and had a lower alcohol intake, and the percentages both of married subjects and of participants with a higher educational level (university) were lower.

**Table 1:** Characteristics of participants in each randomized intervention group

	**MD-EVOO**	**MD-nuts**	**Control diet**
	**(n=1446)**	**(n=1293)**	**(n=1184)**
Age, years	67.1, 6.2	66.7, 6.0	67.4 (6.4)
Male sex, %	47.1	53.5	46.2
Smoking status, %			
Never-smoker	57.7	56.5	58.1
Current smoker	14.5	15.2	14.9
Marital status, %			
Married	80.2	79.9	77.0
Educational level, %			
Primary or lower	76.5	75.2	79.1
University	8.6	8.4	6.3
Disease prevalence, %			
Cancer	2.1	3.2	2.3
Diabetes	52.4	48.1	50.7
Hypertension	80.2	81.1	82.3
Hypercholesterolemia	68.9	69.5	67.7
Parkinson disease	0.3	0.3	0.3
Chronic bronchitis	4.8	4.7	5.4
Fractures	16.1	17.8	18.7
Body mass index, kg/m^2^	29.8, 3.7	29.6, 3.7	30.1 (4.0)
Total energy intake, kcal/day	2317.9, 619.1	2384.1, 611.0	2256.4 (605.8)
Alcohol intake, g/day	10.0, 15.6	11.2, 17.1	9.0 (14.9)
Physical activity during leisure time, METs-min/day	247.4, 243.6	270.9, 258.7	226.9 (261.1)

Abbreviations: *MET* metabolic equivalent, *EVOO* extra virgin olive oil.

We identified 224 new cases of depression during the follow-up period (median 5.4 years). The effect of the interventions on the incidence of depression is shown in Table 2. An inverse association with depression was seen for participants assigned to receive MD-nuts, although this was not significant; multivariate HR = 0.78 (95% CI 0.55 t. 1.10) for the MD-nuts group compared with the control (low-fat) group. When both MDs were merged together and analyzed as a single group, no significant association was found between the MD intervention and depression risk (multivariate HR = 0.85; 95% CI 0.64 to 1.13).

**Table 2:** Risk of incident depression in each randomized group

	**Nutritional intervention**		
	**Control diet**	**MD-EVOO**	**MD-nuts**
Cases	77	88	59
Person-years	6,096	7,715	6,803
Model 1^a^			
HR (95% CI)	1 (ref)	0.85 (0.62 to 1.15)	0.73 (0.52-1.03)
HR (95% CI)^b^	1 (ref)	0.80 (0.60 to 1.05)	
Model 2^c^			
HR (95% CI)	1 (ref)	0.91 (0.67 to 1.24)	0.78 (0.55-1.10)
HR (95% CI)^b^	1 (ref)	0.85 (0.64 to 1.13)^c^	

Abbreviations: *HR* hazard ratio, *MD* Mediterranean diet, *ref* reference group, *EVOO* extra virgin olive oil.

^a^Model 1: adjusted for age, sex, and recruiting center.

^b^Combined MD.

^c^Model 2: model 1 additionally adjusted for body mass index, smoking, physical activity during leisure time, educational level, marital status, alcohol and total energy intake, and presence of any of several diseases at baseline (cancer, diabetes mellitus type 2, hypertension, hypercholesterolemia, fractures, Parkinson disease and chronic bronchitis).

Results from sensitivity analyses are shown in Table 3. In most of the sub-analyses, similar results to those found in the main analyses were obtained, with reductions of around 20 to 30% in the risk of developing depression for those participants assigned to the MD-nuts intervention, although the estimates did not reach statistical significance. However, when the analysis was restricted to participants with DM2, a stronger and significant reduction in the risk of depression was seen for participants assigned to the MD-nuts group compared with those assigned to the control group (41% relative reduction in the risk of developing depression; 95% CI 0.36 to 0.98; *P* = 0.04).

**Table 3:** **Association between nutritional interventions and depression**^**a**^

**Analysis**	**Total, n**	**Cases, n**	**Nutritional intervention, HR (95% CI)**		
			**Control diet**	**MD-EVOO**	**MD-nuts**
Excluding late cases (>6 years of follow-up)	3,893	211	1 (ref)	0.88 (0.64 to 1.21)	0.73 (0.52 to 1.05)
Including only participants with diabetes	1,958	113	1 (ref)	0.71 (0.46 to 1.09)	0.59 (0.36 to 0.98)
Including only participants with hypertension	3,181	181	1 (ref)	0.93 (0.66 to 1.31)	0.79 (0.54 to 1.16)
Including only participants with hypercholesterolemia	2,697	158	1 (ref)	0.93 (0.64 to 1.35)	0.81 (0.54 to 1.21)
Including only participants with obesity	1,781	113	1 (ref)	0.85 (0.55 to 1.31)	0.69 (0.41 to 1.13)
Excluding participants with limiting conditions^b^	3,074	184	1 (ref)	0.81 (0.57 to 1.13)	0.68 (0.46 to 0.99)
Excluding participants with cancer	3,824	216	1 (ref)	0.90 (0.66 to 1.24)	0.79 (0.56 to 1.12)
Excluding participants with potentially missing events^c^	3,867	224	1 (ref)	0.91 (0.66 to 1.24)	0.78 (0.55 to 1.10)
Multiple imputation for missing values	82,383	4,740	1 (ref)	0.95 (0.69 to 1.32)	0.75 (0.52 to 1.07)

Abbreviations: *HR* hazard ratio, *MD* Mediterranean diet, *ref* reference group, *EVOO* extra virgin olive oil.

^a^Adjusted for age, sex, recruiting center, body mass index, smoking, physical activity during leisure time, educational level, marital status, alcohol and total energy intake, and the presence of several diseases at baseline (cancer, diabetes mellitus type 2, hypertension, hypercholesterolemia, fractures, Parkinson disease and chronic bronchitis).

^b^Such as fractures, PD, and chronic bronchitis.

^c^Approaches for dealing with missing data were as follows. We implemented the suggestions given by Groenwold *et al*. [25] to handle potentially missing outcome data in randomized trials. In order to conduct sensitivity analyses on missing outcomes, we considered as missing outcome data the potential occurrence of depression for 59 participants for whom we had no documented incident depression and who were lost to follow-up for 2 years or longer. We conducted two sensitivity analyses: 1) in the complete case analysis we excluded these 59 subjects from the computation of HRs and adjusted the Cox model for all the covariates mentioned in Table 3; 2) in multiple imputations, 20 values were sampled from an estimated uniform distribution (also taking into account the previously mentioned predictors) and imputed for the 59 participants lost to follow-up adding a random term. Hence, 20 datasets with imputed outcomes were created. Each dataset was analyzed using multivariable-adjusted Cox models and, subsequently, the results were pooled by using standard techniques, also taking into account the variation between imputed data sets [24].

Table 4 shows the results from the per-protocol analysis. After 3 years of follow-up, we had complete data from 2,513 participants about their dietary habits and energy intake. Compared to those participants with the lowest adherence to the MD, those with the highest adherence to the MD did not show a significant decrease in the risk of developing depression during the follow-up (multivariate HR = 0.71; 95% CI 0.38 to 1.32).

**Table 4:** Risk of developing depression stratified by level of adherence to Mediterranean diet (per-protocol analysis) after 3 years of follow-up

	**Adherence to MD**			
	**Low: <8 points**	**Medium: 8–9 points**	**High: ≥10 points**	***P*-value for trend**
Median (points)	7	9	11	
Cases, n	14	32	81	
Person-years	1,105	3,059	8,872	
Model 1				
HR (95% CI)	1 (ref)	0.81 (0.43 to 1.51)	0.72 (0.40 to 1.28)	0.26
Model 2				
HR (95% CI)	1 (ref)	0.73 (0.38 to 1.39)	0.71 (0.38 to 1.32)	0.38

Abbreviations: *HR* hazard ratio, *MD* Mediterranean diet, *ref* reference.

Model 1: adjusted for age, sex, and recruiting center.

Model 2: model 1 additionally adjusted for intervention group, for smoking, educational level, marital status, and the presence of any of several diseases at baseline, and body mass index, physical activity during leisure time, alcohol intake (except wine), and total energy intake after 3 years of follow-up.

## Discussion

We did not find a significant decrease in depression risk among participants at high risk of CVD assigned to MD supplemented with either nuts or EVOO in this randomized controlled primary prevention trial. However, when the analysis was restricted to subjects with DM2, participants assigned to MD-nuts had a 40% reduction in depression risk compared with the control group, which was significant.

To our knowledge, this is the first randomized field trial that has ascertained the effect of an intervention with an overall dietary pattern on depression risk in adults. Only a few prospective observational studies have inversely related healthy dietary patterns to the risk of developing adult depression [7-15]. Although some of these studies were based only on cross-sectional assessments [8,9,11-13], their results were consistent with those obtained after several years of follow-up in prospective cohorts [7,10].

Regarding the MD, a very recent cohort study, the Australian Longitudinal Study on Women’s Health [14], found that women in the highest quintile of adherence to a ‘Mediterranean-style’ diet were 37% less likely to report depressive symptoms after 3 years of follow-up, (adjusted odds ratio (OR) = 0.63, 95% CI 0.47 to 0.85) compared with those in the lowest quintile of adherence. Similar results were previously obtained in the SUN (Seguimiento Universidad de Navarra) Project [15] in which participants with the highest adherence to the MD had a significant reduction in the risk of developing depression (comparison of fifth versus first quintile: adjusted HR = 0.58; 95% CI 0.44 to 0.77).

Nevertheless, the available evidence is still sparse and not definitive. Moreover, interpretation of findings resulting from observational studies demand caution [3]. Most of the studies had a cross-sectional design [8,9,11-13], which is a weak design for inferring cause and effect relationships that can only be suggested. In such studies, exposure is ascertained simultaneously with disease and, therefore, an alternative interpretation of the results could be made as a consequence of reverse causation bias; for example, that depression may lead to poorer dietary habits [27]. These large studies generally need the use of food frequency questionnaires to collect information on dietary factors, and although such questionnaires have been customarily used and generally have been validated, it is known that they have some potential for misclassification bias. Finally, it is necessary to take into account the possibility of residual confounding.

By contrast, findings from observational studies can be supported by some biochemical and physiological mechanisms that may be implicated in depression risk and that are also intimately related to dietary factors. Examples include low-grade systemic inflammation and endothelial and metabolic disturbances, which may be present in patients with depression [28-31]. Indeed, a large number of clinical trials including the PREDIMED study and some observational studies have reported an inverse association between adherence to a MedDiet pattern and the levels of inflammatory, metabolic, or endothelial biomarkers [32-35]. The presence of inflammatory processes and endothelial dysfunction compromise the production and secretion of brain-derived neurotrophic factor (BDNF), a peptide implicated in synaptic plasticity and neuronal survival, and whose levels are decreased in patients with depression [36]. In a previous sub-analysis of the PREDIMED trial, conducted by our group in the PREDIMED-NAVARRA center, significantly higher plasma BDNF levels were seen for patients with depression assigned to the MD-nuts compared with those assigned to a control diet [37].

Our results indicate that adherence to a Mediterranean dietary pattern supplemented with nuts could be particularly important to prevent depression among participants with DM2. The association between obesity, DM2, metabolic syndrome (MetS), and depression has been suggested in several studies [38-40]. Metabolic disturbances/dysregulation of markers such as insulin, leptin, glucose [30,31], or tryptophan/serotonin [41,42] could explain the link between obesity, DM2, and depression. In fact, in the Whitehall II cohort study, low insulin secretion was associated with an increased risk of developing depressive symptoms [30]. Recent studies have also reported an increased risk of depressive symptoms associated with higher levels of leptin [43], especially in the presence of abdominal obesity [31,44]. The association of leptin with depression could be explained not only by its metabolic properties but also by its neurobiological activity, as leptin is able to affect neuroprotection, cognition, and mood in the hippocampus, the cortex, and other brain areas [45]. Moreover, in several studies, hyperleptinemia and insulin resistance, which can be present in obesity, MetS, and DM2 have been also linked to endothelial dysfunction or inflammation processes [46,47], conditions also present in depression. Not only metabolic disturbances but also inflammatory markers have been recently associated with depressive symptoms in participants with diabetes from the SEARCH for Diabetes in Youth cohort study [48].

Interestingly, within the PREDIMED trial, several sub-analyses support this hypothesis. After 3 months of follow-up, participants assigned to the MD + nuts exhibited significant reductions in fasting glucose and insulin levels and in Homeostasis Model Assessment index compared to those assigned to the control diet [49]. Moreover, a significant reversion of MetS was seen in the MD-nuts group after 1 year of intervention; in this group, the OR for reversion of MetS was 1.7 (95% CI=1.1 to 2.6) compared with the control diet group [33].

Moreover, in a recent clinical trial based on a sample of patients with MetS assigned to receive a control diet or a diet enriched in mixed nuts for 12 weeks, two intermediate urinary metabolites of the tryptophan pathway (which leads to biosynthesis of serotonin and melatonin from tryptophan) were identified: N-acetylserotonin sulfate and hydroxyindoleacetic acid [50]. These metabolites are considered urinary markers of nut intake, as walnuts are one of the most important dietary sources of serotonin [51]. However, the authors pointed out that the presence of these urinary metabolites could be due both to a high intake of walnuts and to a high endogenous serotonin turnover following the intake of these food items. Thus, such metabolites could be markers of the effect of the global dietary intervention, instead of being markers of intake [50].

We did not find a significant effect on depression risk for adherence to the MD supplemented with EVOO in the overall sample. However, when the analyses were restricted to those participants with DM2. the relationship was strengthened. The effect on depression of the intervention with MD supplemented with EVOO for patients with DM2, although non-significant, was similar to that obtained for patients with DM2 in the analysis of the effect of MD on risk of CVD in the PREDIMED framework [16]. As mentioned above, several physiological processes may be responsible for the link between depression and cardiovascular and metabolic disorders [28-31]. In fact, a beneficial role of supplementation with a Mediterranean diet enriched with olive oil in various processes such as oxidative stress, inflammation, lipid metabolism, or weight regulation for patients with DM2 or multiple sclerosis has been described in several epidemiological studies [52-54].

Although our results indicate a possible beneficial role of MD supplemented with nuts (compared with a low-fat diet) in the prevention of depression, with a risk reduction of around 25%, the results were not statistically significant. There are several reasons that may explain the lack of statistical significance. First, the number of new cases was not large. In the report from the SUN cohort relating the Mediterranean diet with depression, 480 new cases of depression were identified [15], whereas in the present study, the number of new cases was less than half of that the SUN cohort. Second, observational studies generally compare extreme quintiles or quartiles of adherence to a dietary pattern, therefore, high between-subject variability in adherence allows for comparison of extremes in exposure and guarantees that a strong effect can be detected. By contrast, in the present study, the variability in adherence to the MD between the Mediterranean and control groups was relatively small. In fact, after 6 years of follow-up, the participants assigned to the control group showed a mean adherence to the MD of 9 points (up to 14 points maximum), whereas the mean adherence within the groups assigned to both MDs was 10.5 points [16].

Moreover, in intervention trials, the degree of change in the dietary habits of participants is always suboptimal, because of the lack of compliance with the intended intervention of some participants [19]. In fact, the control group in the PREDIMED trial was assigned a healthy dietary pattern recommended by the American Heart Association to prevent CVD [18], and also they tended at baseline to be on a dietary pattern that was similar to the Mediterranean diet. This reality suggests that there would be even a potentially greater benefit of the Mediterranean diet if it were compared to a typical (and unhealthy) Western diet [16]. Third, the PREDIMED trial was not designed to study depression as a primary end-point; the primary end-point was in fact a composite of cardiovascular clinical events. Therefore, it is likely that some degree of misclassification in the ascertainment of depression cases may have happened. However, we took care to ensure that all included cases had received a medical diagnosis of depression (or were under antidepressant treatment), and that they were not prevalent cases at baseline. In fact, we excluded all participants with a short follow-up period (the first 3 years) in order to ensure that reverse causation could not explain the reported results.

Although in the PREDIMED trial, follow-up data were available for 97% of the sample, it is important to note that the time each participant remained in the trial was variable because centers started to recruit participants from 2004 to 2006, many centers continued to recruit participants until 2009, and the trial ended in 2010. Thus, to obtain a more homogeneous period of follow-up in all participants, we carried out our analyses only with those participants with at least 3 years of follow-up. Usually, to minimize the influence of the presence of undiagnosed disease at baseline, a methodological approach in cohort studies has been to exclude cases occurring during the first 3 [55] or even the first 5 years of follow-up [56]. We chose a 3-year period because we believed it likely that participants with undetected (sub-clinical) depression at baseline might receive a delayed diagnosis of depression during the first, second, or even third year of follow-up. However, we considered that in participants with true but undiagnosed depression at baseline, it was unlikely that their depression would remain undetected during the first 3 years of follow-up and that they might yet receive a delayed diagnosis only during the fourth year of follow-up or later.

One of the main problems of a nutritional intervention trial is the variability in the compliance of participants with the intended dietary intervention. To address this issue, we carried out a per protocol approach as an ancillary analysis to compare the results, taking into account what the participants really did. The category of highest adherence to the MD after 3 years of intervention exhibited the lowest risk of depression. This is consistent with the results obtained in the ITT analyses. However, in both case,s the lack of statistical significance is probably attributable to the small number of cases included.

Finally, the highest retention rates were seen in the MD groups, and the lowest retention rate was seen in the control (low-fat diet) group. This high retention rate in the two MD group by be partly attributable to the free provision of specific food items (EVOO and nuts). In additon, the palatability of the Mediterranean diet has been identified as a key factor in its higher compliance [57]. In general, the research group was able to obtain nearly complete follow-up for the main outcomes because participants represented a stable and well-defined population regularly attending their general practitioners. In addition, a comprehensive search for events was performed yearly through review of all the medical records of participants in all the university hospitals of the area in which the respective recruiting center was located. Nevertheless, 59 participants without a diagnosis of incident depression could not be contacted for at least 2 years (22 participants assigned to the low-fat group, 19 to the MD-nuts and 18 to the MD+EVOO). Thus, differential misclassification bias in the outcome is not a very likely possibility. Moreover, when the analyses were repeated imputing the missing values for subjects lost to follow-up, the results did not change substantially.

## Conclusions

In conclusion, results from this analysis are suggestive of a beneficial effect of a long-term intervention with a Mediterranean diet on depression for patients with DM2. Nevertheless, to definitely assess the role of Mediterranean diet in the prevention of depression, longer follow-up of this trial and further experimental investigations are needed.

## Abbreviations

BDNF: Brain-derived neurotrophic factor; BMI: Body mass index; CI: Confidence interval; CVD: Cardiovascular disease; DALY: Disability-adjusted life years; DM2: Diabetes mellitus type 2; EVOO: Virgin olive oil; HDL: High-density lipoprotein; HR: Hazard ratio; HTA: Hypertension; LDL: Low-density lipoprotein; MD: Mediterranean diet; MetS: Metabolic syndrome; OR: Odds ratio; PD: Parkinson disease; PREDIMED: Prevención con Dieta Mediterránea; SUN: Seguimiento Universidad de Navarra; YLD: Years lost to disability.

## Competing interests

RE reports serving on the board of and receiving lecture fees from the Research Foundation on Wine and Nutrition (FIVIN); serving on the boards of the Beer and Health Foundation and the European Foundation for Alcohol Research (ERAB); receiving lecture fees from Cerveceros de España and Sanofi-Aventis; and receiving grant support through his institution from Novartis. JSS reports serving on the board of and receiving grant support through his institution from the International Nut and Dried Fruit Council; receiving consulting fees from Danone; and receiving grant support through his institution from Eroski and Nestlé. FA reports receiving payment for the development of educational presentations from Menarini and AstraZeneca. XP reports serving on the board of and receiving grant support through his institution from the Residual Risk Reduction Initiative (R3i) Foundation; serving on the board of Omega Fort; serving on the board of and receiving payment for the development of educational presentations, and receiving grant support through his institution, from Ferrer; receiving consulting fees from Abbott; receiving lecture fees, and receiving grant support through his institution, from Merck and Roche; receiving lecture fees from Danone and Esteve; receiving payment for the development of educational presentations from Menarini; and receiving grant support through his institution from Sanofi-Aventis, Kowa, Unilever, Boehringer Ingelheim, and Karo Bio. RMLR reports serving on the board of and receiving lecture fees from FIVIN; receiving lecture fees from Cerveceros de España; and receiving lecture fees and travel support from PepsiCo. ER reports serving on the board of and receiving travel support, and receiving grant support through his institution, from the California Walnut Commission; serving on the board of the Flora Foundation (Unilever); serving on the board of and receiving lecture fees from Roche; serving on the board of and receiving grant support through his institution from Amgen; receiving consulting fees from Damm and Abbott; receiving consulting fees and lecture fees, and receiving grant support through his institution, from Merck; receiving lectures fees from Danone, Pace, AstraZeneca, and Rottapharm; receiving lecture fees and payment for the development of educational presentations, and receiving grant support through his institution, from Ferrer; receiving payment for the development of educational presentations from Ricordati; and receiving grant support through his institution from Sanofi-Aventis, Takeda, Daiichi Sankyo, Nutrexpa, Feiraco, Unilever, and Karo Bio. LSM reports serving on the boards of the Mediterranean Diet Foundation and the Beer and Health Foundation. No other potential conflict of interest relevant to this article was reported by any of the authors.

## Authors’ contributions

All authors had full access to all of the data in the study and take responsibility for the integrity of the data and the accuracy of the data analysis. All authors participated in the acquisition of data. ASV, MAMG, and LSM were responsible for the study concept and design; MAMG and LSM supervised the study; ASV and AG performed the statistical analysis; ASV, MAMG, and LSM interpreted the data; ASV and MAMG drafted the first version of the manuscript; all other authors contributed to subsequent drafts and the final paper, and made important comments for intellectual content. All authors read and approved the final manuscript.

## Pre-publication history

The pre-publication history for this paper can be accessed here:

http://www.biomedcentral.com/1741-7015/11/208/prepub
